# Single Nucleotide Polymorphisms (SNPs) Reveal Sibship Among Founders of a Bangladeshi Rohu (*Labeo rohita*) Breeding Population

**DOI:** 10.3389/fgene.2019.00597

**Published:** 2019-06-19

**Authors:** Matthew Gray Hamilton, Wagdy Mekkawy, Andrzej Kilian, John A. H. Benzie

**Affiliations:** ^1^WorldFish, Penang, Malaysia; ^2^Animal Production Department, Faculty of Agriculture, Ain Shams University, Cairo, Egypt; ^3^Diversity Arrays Technology Pty Ltd., (DArT P/L), University of Canberra, Bruce, ACT, Australia; ^4^School of Biological, Earth and Environmental Sciences, University College Cork, Cork, Ireland

**Keywords:** parentage assignment, genomic relationship, additive genetic relationship, carp, cyprinidae, genotyping-by-sequencing, single nucleotide polymorphism, silicoDArT marker

## Abstract

Rohu (*Labeo rohita*) is a significant freshwater aquaculture species with approximately 1.8 Mt produced annually. Fin clips obtained from the founders of a newly established Bangladesh-based breeding population (∼140 fish from each of the Halda, Jamuna, and Padma rivers) were used to identify 9157 SNPs and 14 411 silicoDArT markers using the Diversity Arrays Technology (DArT) genotyping-by-sequencing platform known as DArTseq. After quality control, 1985 SNPs were retained and used to examine population structure within and among river systems. Examination of genomic relationships revealed evidence of full- and half-sibling relationships among founders. Accordingly, sibship and dummy parents were assigned within each river population using a maximum likelihood approach with COLONY software. Founders that had no dummy parents in common were then identified for population genetic analyses. Only 40 unique dummy parents and 17 founders with no common dummy parents were identified from the Halda river, compared with 206 (96) from the Jamuna and 184 (83) from the Padma. Overall pairwise F_ST_ estimates among rivers were low (<0.005) and the optimum number of clusters using unsupervised K-means clustering was one, indicating little genetic divergence among the river populations in our SNPs. These results suggest that observed sibship among founders should be accounted for in future pedigree-based analyses and it cannot be assumed that fertilized spawn collections are representative samples of river populations.

## Introduction

Rohu (*Labeo rohita* Hamilton) is a member of the Indian major carps and has a natural distribution encompassing rivers in Bangladesh, Myanmar, Nepal, and Pakistan, as well as the tributaries and branches of the Ganges river in northern India (Jhingran and Pullin, 1985). It is a globally significant freshwater aquaculture species with approximately 1.8 Mt produced annually (FAO, 2017).

In Bangladesh, rohu represents the most abundantly cultured carp species (DOF, 2017) but a lack of a genetic improvement program and suboptimal genetic management of hatchery broodstock has resulted in the widespread dissemination of seed exhibiting poor performance attributed to inbreeding, negative selection and interspecific hybridization (Penman et al., 2005; Das Mahapatra et al., 2016; Khan et al., 2018). To address these issues, in 2012, fish were collected as fertilized spawn from three Bangladeshi rivers – the Halda, Jamuna, and Padma – as part of a USAID funded project implemented by WorldFish known as Aquaculture for Income and Nutrition (AIN). The fertilized spawn was then reared and distributed to hatcheries in an effort to improve the genetic quality of rohu seed produced in Bangladesh (Keus et al., 2017).

Of the rivers from which fertilized spawn was collected as part of the AIN project, the Padma is the largest. It represents largest branch of what is known as the Ganges river in India. The Jamuna is also part of a major river system and encompasses the lower reaches of the Brahmaputra river, which ultimately flows into the Padma. The Halda river, in comparison, is relatively small and is hydrologically and geographically isolated from the Jamuna and Padma rivers. All three rivers are important natural breeding grounds for rohu and have historically been important sources of rohu seed for aquaculture (Penman et al., 2005; Khan et al., 2018).

In 2013, fish reared as part of the AIN project were identified as an appropriate source of founders for the establishment of a rohu breeding population. In 2014, a base population for genetic improvement was established through the mating of founders sourced from the AIN collections (Keus et al., 2017). The WorldFish rohu breeding population has subsequently been managed as discrete generations with a generation interval of 2 years.

DArTseq is a high-throughput genotyping-by-sequencing (GBS) technology that uses a combination of genome complexity reduction methods implemented by Diversity Arrays Technology Pty Ltd. (DArT) (Kilian et al., 2012). DArTseq generates data for two biallelic markers types – dominant “silicoDArT” markers (i.e., scored as either present or absent) and co-dominant single nucleotide polymorphisms (SNPs; scored as one of two homozygous states or as heterozygous). DArTseq has been applied to a wide range of species and applications including the study of inter- and intra-specific genetic diversity and relationships, genetic mapping, genome wide association studies, and genomic selection (Egea et al., 2017; Edet et al., 2018; Nguyen et al., 2018a,b; Hamilton et al., 2019b). Past studies in rohu using Randomly Amplified Polymorphic DNA (RAPD) and microsatellite markers, generally revealed low levels of molecular marker differentiation between rohu river populations, with all but one reported statistically significant estimate of F_ST_ being less than 0.043 (Islam and Alam, 2004; Alam et al., 2009; Sahoo et al., 2014; Ullah et al., 2015; Qadeer and Abbas, 2017). A number of these studies included samples from the Halda, Jamuna, and Padma rivers (Islam and Alam, 2004; Alam et al., 2009; Ullah et al., 2015). In addition, Ullah et al. (2015) identified a number of half-sibling and full-sibling relationships between hatchery broodstock collected as spawn from the Halda, Jamuna, and Padma rivers. In this context, the primary objectives of this study were to (i) identify SNPs and silicoDArT markers for rohu, (ii) estimate population genetic parameters using DArTseq SNP, (iii) examine population structure within and among the three sampled Bangladeshi rivers using DArTseq SNP, and (iv) validate, or otherwise, the assumption that the founders of the WorldFish genetic improvement population were unrelated.

## Materials and Methods

In 2012, fertilized rohu spawn was collected using commercial spawn harvesting methods (Rahman, 2008) from two locations in each of three major river systems in Bangladesh; the Halda, Jamuna and Padma (refer to Khan et al., 2018 for details). Eggs were then hatched and fish reared in separate ponds according to their river of origin. At 2 years of age, approximately 140 individuals from each river were mated to form a base population for genetic improvement (Keus et al., 2017). All founders were fin-clipped, as part of the routine husbandry of the population. Fish were anesthetized, with clove oil, prior to the removal, with scissors, of an approximately 2-mm-wide fin-clip. Fish were then placed in recovery tanks for monitoring and only released back into ponds once they had satisfactorily recovered from anesthesia. Fish in the breeding population are managed in accordance with the Guiding Principles of the Animal Care, Welfare and Ethics Policy of the WorldFish Center (Worldfish, 2004).

For the purpose of the current study, archived fin-clip samples of all but four founders, for which fin-clips were not available, were genotyped using the DArTseq platform in 2016. The laboratory procedures and analytical pipelines outlined in Appendix S1 of Lind et al. (2017) were followed, with the exception that the complexity reduction method involved a combination of PstI and SphI enzymes (SphI replacing HpaII used in Lind et al., 2017). Briefly, the PstI-compatible adapter was designed to include Illumina flow-cell attachment sequence, sequencing primer sequence and “staggered,” varying length barcode region, similar to the sequence reported by Elshire et al. (2011). Reverse adapter contained flow-cell attachment region and SphI-compatible overhang sequence. Only “mixed fragments” (PstI-SphI) were effectively amplified in 30 rounds of PCR. After PCR, equimolar amounts of amplification products from each sample of the 96-well microtiter plate were bulked and applied to c-Bot (Illumina) bridge PCR followed by sequencing on Illumina Hiseq2500. The sequencing (single read) was run for 77 cycles.

Sequences generated from each lane were processed using proprietary DArT analytical pipelines which have been deployed over the last decade to produce marker data for thousands of species. In the primary pipeline the fastq files were first processed to filter away poor quality sequences, applying more stringent selection criteria to the barcode region compared to the rest of the sequence. In that way the assignments of the sequences to specific samples carried in the “barcode split” step were very reliable.

Filtering was performed on the raw sequences using the following parameters: “Min Phred pass score 30, Min pass percentage 75” (Barcode region) and ‘Min Phred pass score 10, Min pass percentage 50′ (Whole read). The mean number of sequences identified per sample was approximately 2.45 million. For SNP calling, all tags from all libraries included in the DArTsoft14 analysis were clustered using DArT PL’s C++ algorithm at the threshold distance of three, followed by parsing of the clusters into separate SNP loci, checking the balance of read counts for the allelic pairs was within a small range (up to 4-fold difference). The co-efficient of variation in sequences per sample was around 8% and variation in counts among samples was low, therefore selecting markers based on average read depth for the whole experiment was deemed sufficient. This assertion was tested by proving Mendelian behavior of markers called by DArTsoft14 from DArTseq libraries in over 1000 controlled genetic crosses. Rejection of very large clusters (e.g., >100) and checking for feasible SNP-reference allele proportions facilitated selection of true allelic variants from paralogous sequences. In addition, multiple samples were processed from DNA to allelic calls as technical replicates and scoring consistency was used as the main selection criteria for high quality/low error rate markers. Only markers with average reproducibility of 95% were accepted, although 99% of markers were completely concordant among technical replicates. Confidence in calling quality was supported by high average read depth per locus, high marker score reproducibility and high call rate percentages (Table 1). DArTseq outputs are available upon request at https://doi.org/10.7910/DVN/PWC5EY (Hamilton et al., 2019b). Data for 10 fin-clipped individuals (samples BFA4815, BFA4644, BFA4570, BFA4541, BFA4960, BFA4491, BFA4383, BFA4672, BFA4760, and BFA4371), that were not breeding program founders, were excluded from the dataset prior to further analysis.

**Table 1:** Summary statistics for genomic markers identified by DArTseq.

	Marker Type		
	silicoDArT	SNP	SNP (post quality control)
Number of markers	14 411	9157	1985
Fragments sequenced containing one marker	14 409	6346	1985
Fragments sequenced with multiple markers	1	1277	0
Unique fragments	14 410	7623	1985
Average fragment length (base pairs)	59.8 (0.12)	65.3 (0.11)	65.1 (0.21)
Fragment length minimum (base pairs)	20	20	20
Fragment length maximum (base pairs)	69	69	69
Polymorphic information content	0.16 (0.001)	0.20 (0.002)	0.26 (0.002)
Call rate	0.94 (0.001)	0.69 (0.004)	0.99 (0.0002)
Reference read depth	43.6 (0.33)	28.9 (0.35)	39.0 (0.86)
SNP read depth	NA	19.7 (0.23)	27.0 (0.58)
Reproducibility^∗^	0.998 (0.0001)	0.995 (0.0001)	0.990 (0.0003)
Avg. missing data per individual (%)	5.4 (0.36)	31.0 (0.09)	0.56 (0.07)

*Standard errors are in parentheses.^∗^ Reproducibility is the proportion of technical replicate assay pairs for which the marker score was consistent.*

Analyses of genomic data were primarily conducted in R (version 3.4.4; R Core Team, 2018). DArT SNP data were initially converted to a “genlight” R object, which allows the storage of SNP data in a compact form (Jombart and Ahmed, 2011), using the “new” function (R Core Team, 2018). To retain SNPs of high quality, in approximate linkage equilibrium and informative for analyses, quality control procedures were implemented. Firstly, SNPs with an observed minor allele frequency (MAF) ≤0.05 or a rate of missing observations ≥0.05 were excluded (Supplementary Figure S1.1). Secondly, to avoid the inclusion of multiple physically linked SNPs from any one DNA sequence/fragment, only one random SNP was retained from each. Thirdly, pairwise squared correlations of genotypic allele counts were computed as a measure of linkage disequilibrium (LD). To prune SNPs for pairwise LD, a random SNP from the pair with the highest r^2^ was then excluded iteratively until all pairwise r^2^ values were ≤0.2 (Hodoğlugil and Mahley, 2012). Finally, filtering of SNPs for deviations from Hardy-Weinberg equilibrium (HWE) was conducted using data from a subset of fish in which close relatives were removed. Close relatives were identified in a preliminary analysis (method outlined below) and were removed to reduce the risk of false identification of SNPs exhibiting genotyping problems (see Wang, 2018). Deviation from HWE in each SNP and sampled population was tested using the “hw.test” function of the “pegas” package (Version 0.10; Paradis, 2010) after data conversion using the “df2genind” function (version 2.1.1; Jombart and Ahmed, 2011). Single nucleotide polymorphisms that significantly deviated from HWE in any sampled population were excluded (classical χ^2^ test; *P* < 0.05 after Dunn–Šidák correction).

Post SNP quality control, a genomic relationship matrix (**G**) was generated according to the first method proposed by Vanraden (2008):

$$G=\frac{\mathrm{ZZ'}}{2\sum {\text{p}}_{\text{i}}(1-{\text{p}}_{\text{i}})}$$

Where **Z** = **M**–**P**, **M** is a matrix of dimensions equal to the number of individuals by the number of loci and specifies which marker alleles each individual inherited (elements are set to -1, 0, and 1 for the homozygote, heterozygote, and other homozygote), **P** is a matrix that specifies allele frequencies expressed as a difference from 0.5 and multiplied by 2, and p_i_ is the frequency of the second allele at locus i. This method was implemented using code from Gondro (2015), page 133 modified to replace missing observations in each SNP (representing only 0.56% of all observations), according to the average of the observed allele frequency. Subsequent clustering of genomic relationships, according to the “Ward2” algorithm implemented in the “hclust” function (Murtagh and Legendre, 2014), revealed evidence of full-sib and half-sib relationships among founders (i.e., the presence of excessive close relatives; Wang, 2018). Full- and half-sibling relationships among founders (and dummy parents) were thus assigned, using a maximum-likelihood approach with COLONY software (version 2.0.6.4; Jones and Wang, 2010). A modified pedigree for founders was then constructed assuming COLONY-derived dummy parents were unrelated. COLONY inputs were generated separately for each sampled river population assuming SNPs were unlinked (i.e., on separate chromosomes), using the default settings of the “write_colony” function of the “radiator” package (version 0.0.11; Gosselin, 2017) except that allele frequencies were set to update. Errors in the “write_colony” output were observed and corrected manually – specifically, the seed for the random number generator, the number of offspring with a known father and mother, and the output file name were manually entered into “Colony2.dat” files. To reduce computation time, while maintaining sufficient SNP for sibship assignment, only those SNPs with a MAF greater than 0.2 (1017 for Halda, 1037 for Jamuna, and 1040 for Padma) were retained in COLONY analyses.

To mitigate the effects of sampling excessive close relatives on estimates of population genetic parameters (Wang, 2018), putatively unrelated individuals were identified using the COLONY sibship assignments. These individuals were identified by (i) generating the additive relationship matrix (**A**) from the COLONY-derived pedigree using the “makeA” function of the “nadiv” package (version 2.16.0.0; Wolak, 2012); (ii) listing individuals that were unrelated (a_ij_ = 0) to other individuals in **A** and then removing these individuals from **A**; (iii) appending to the list generated in step ii the individual remaining in **A** with the lowest average relationship with the other individuals and then removing this individual and its relatives (a_ij_ > 0) from **A**; and (iv) iteratively repeating step iii until no individuals remained in **A** (see Supplementary Material 2 for a worked example). A small number of pairwise genomic relationships between founders purportedly from different rivers were very strong. These were attributed to labeling or fish-management mistakes (e.g., fish may have jumped, or been mistakenly transferred, over physical barriers) and data from associated animals were omitted in subsequent analyses (10 fish from the Padma and 13 from the Jamuna).

For each sampled river population, **G** matrices were generated using observed allele frequencies from founders with no COLONY-assigned dummy parents in common. To validate the COLONY-derived pedigree, **A** matrices for each river were then compared with **G** matrices.

Observed (H_obs_) and expected (H_exp_) heterozygosities by SNP were estimated, for each sampled population, using the “summary” function of the “adegenet” package. The significance of pairwise population differences in mean H_exp_ were estimated using the “Hs.test” (n.sim = 999) functions of the “adegenet” package. The significance of the differences between H_obs_ and H_exp_ within rivers were tested with paired *t*-tests. Differences in allelic richness and private allelic richness among sampled populations were compared using the rarefaction method, implemented in ADZE (Szpiech et al., 2008). Pairwise overall Wright (1965) F_ST_ values between populations were estimated using the default settings of the “fst_WC84” function of the “assigner” package (version 0.5.0; Weir and Cockerham, 1984; Gosselin et al., 2016), after data conversion using the “tidy_genomic_data” function of the “radiator” package. The 95% confidence intervals for the overall F_ST_ values were also estimated using “fst_WC84” (bootstrapping with 2000 iterations). Analysis of molecular variance (AMOVA) was conducted using the “poppr.amova” function of “poppr” (version 2.7.1; Kamvar et al., 2015). Default settings were used except that variances within individuals were not calculated (within = FALSE), the Hamming distance matrix was computed [dist = bitwise.dist(x)] and the missing data cutoff was set to 10% (cutoff = 0.1). Data was converted for AMOVA using the “as.genclone” function, defining population of origin as the only stratum. Unsupervised (*K*-means) clustering was then undertaken to investigate the possibility that a population structure, other than the predetermined structure (i.e., river of origin), might better fit the data. The “adegenet” package was adopted for this purpose using the “find.clusters” function (default settings except that max.n.clust = 20 and n.start = 1000) and the output of principal component analyses (PCA) conducted using the “glPca” function (default settings except that nf = 500). The optimum number of clusters was identified as that with the minimum Bayesian Information Criterion (BIC).

## Results

From DArTseq-generated sequences (i.e., fragments), 9157 SNPs and 14 411 silicoDArT markers were identified (Table 1). After quality control, 1985 SNPs were retained for analysis.

**FIGURE 1:**
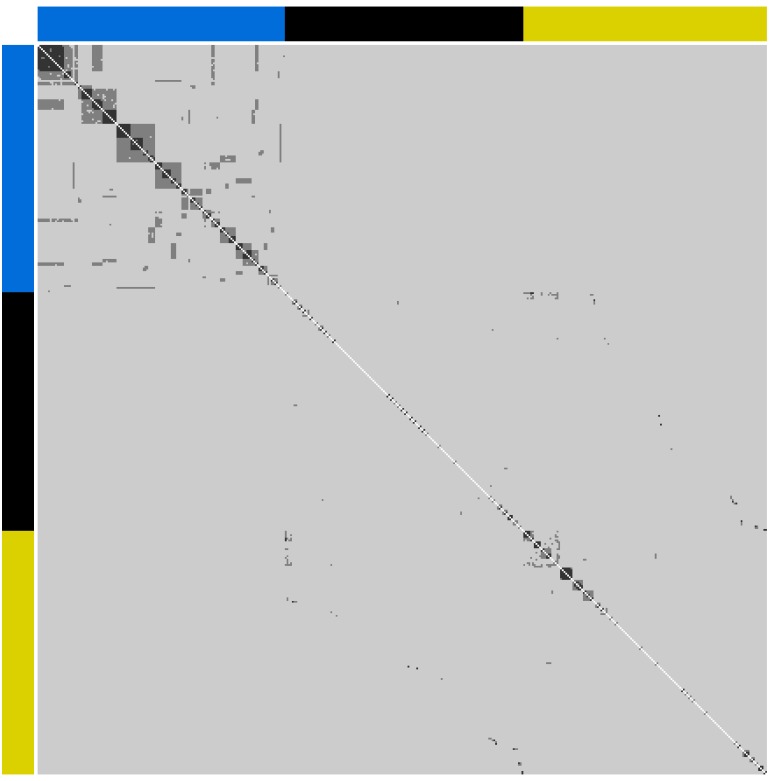
Heatmap of pairwise genomic relationships (<0.156 light gray, 0.156 to 0.370 dark gray and >0.370 black) between individuals from the Halda (top-left; blue bar), Jamuna (middle; black bar) and Padma (bottom right; yellow bar).

Visualization of **G**, computed using observed allele frequencies from all founders from all rivers, as a heatmap (Figure 1) revealed (i) the presence of putative half- and full-sib relationships among founders within rivers and (ii) the presence of strong pairwise relationships between founders purportedly from different rivers. Subsequent sibship assignment with COLONY, indicated that the progeny of only 40 parents contributed to the Halda river breeding population founders, compared with 206 from the Jamuna and 184 from the Padma (Supplementary Figures S1.2–S1.4). Accordingly, only 17 founders with no dummy parents in common were identified from the Halda, compared with 96 from the Jamuna and 83 from the Padma.

Comparison of COLONY-derived **A** matrices with **G** matrices, computed separately for each river using observed allele frequencies from founders with no COLONY-assigned dummy parents in common, revealed few inconsistencies (compare parts c and d in Supplementary Figures S1.2–S1.4). Most notable of these was what appeared to be distant relationships among some Padma river founders in the **G** matrix, which were not evident in the corresponding **A** matrix (Supplementary Figure S1.4a top left). This discrepancy highlights a limitation of COLONY in that it assigns sibship but does not assign more distant relationships. Despite this limitation, the COLONY-derived pedigree undoubtedly represents a closer approximation of reality than the default assumption that founders are unrelated.

Fish sampled from the Halda population had the greatest number of fixed loci prior to, and after, SNP quality control and the removal of putatively related or erroneous individuals, most likely reflecting the small number of founders with no parents in common from this river. Population mean expected heterozygosities were 0.312 (Halda), 0.319 (Jamuna), and 0.317 (Padma) and no significant difference were detected between populations (*P* > 0.117). Consistent with the removal of SNPs deviating from HWE during quality control, differences between mean H_exp_ and H_obs_ were small within populations [observed heterozygosities were 0.313 (Halda), 0.308 (Jamuna) and 0.312 (Padma)], albeit significantly different from zero in the case of the Jamuna and the Padma (*P* < 0.001). Rarefaction analysis revealed no substantive differences in either allelic richness or private allelic richness among rivers (Supplementary Figure S1.5).

The first principal component explained only 1% of the total variance and the three river populations were not clearly distinguishable from each other with respect to the three most important principle components (Supplementary Figure S1.6). Overall multi-locus pairwise estimates of Wright (1965) F_ST_ were also low (<0.005; Supplementary Table S1.1), further indicating little divergence in our SNPs among river populations. Furthermore, variation among populations, although significantly different from zero (*P* < 0.001), represented less than 0.2% of the total molecular marker variance in AMOVA. In addition, unsupervised *K*-means clustering revealed an essentially linear increase in the BIC from 1 to 20 clusters (*K*), indicating the optimum number of clusters to be one and providing further evidence of a lack of substantive genetic structure within and among the sampled populations, once putative siblings had been purged from the data.

## Discussion

The high degree of sibship among rohu breeding population founders observed in this study provides evidence that fertilized spawn samples are not necessarily representative of river populations. This has implications for the management of river-sourced hatchery broodstock (Keus et al., 2017), pedigree-based analyses and inbreeding control in the WorldFish breeding population (Fernández et al., 2014), sampling strategies in future studies, and the interpretation of past population genetic studies (Peterman et al., 2016; Wang, 2018).

Samples of populations taken in early life stages of highly fecund species, including rohu (Ullah et al., 2015), have previously been shown to be prone to the over representation of siblings (Hansen et al., 1997; Goldberg and Waits, 2010; Whiteley et al., 2012; Peterman et al., 2016; Hamilton et al., 2019a). This phenomenon results from the practice of sampling individuals in their early life stages from a limited area over a limited timeframe. In the case of highly fecund species such sampled individuals, although large in number, may represent the progeny of a small number of parents – conceivably just two – that mated in the vicinity at the time of sampling. Such samples thus represent neither a random nor a representative sample of the population as a whole.

In the absence of access to genetically- improved broodstock, Bangladeshi hatcheries currently relying on broodstock collected as spawn from rivers (Keus et al., 2017) should aim to maximize the number of parents contributing to the collected spawn, so as to avoid siblings in broodstock and minimize inbreeding in seed sold for grow out. To this end, spawn should be collected at the peak of the spawning season from stretches of river in which rohu is prevalent, and obtained from multiple locations and/or collection events.

Despite the high level of sibship among Halda river founders of the WorldFish rohu breeding population, 196 (of a total of 420) founders with no dummy parents in common were identified from the three sampled rivers, representing a sizable base population for breeding purposes (Gjedrem and Baranski, 2009). Indeed, the average pedigree-based relationship among founders (i.e., the average of **A**) was 0.0078 (0.0054 for off-diagonals), within the level that would generally be deemed an acceptable increase in the average relationship per generation in a closed breeding population. An increase in average relationship of 0.0078 per generation equates to a future increase in inbreeding per generation (ΔF) of 0.0039 (Meuwissen, 1997) and an effective population size (Ne) of 128, where Ne = 1 / (2ΔF) (Meuwissen and Woolliams, 1994). Accordingly, the unforeseen level of sibship among founders of the, now closed, WorldFish rohu breeding population is unlikely to have a major impact on future parent selection, mating decisions, rates of inbreeding or rates of genetic gain.

The low overall pairwise F_ST_ estimates among rivers, and lack of evidence for genetic structure within or among rivers based on unsupervised *K*-means clustering, is indicative of no or low levels of divergence in our SNPs among river populations. Previous estimates of pairwise F_ST_ between rivers for rohu, using a range of non-SNP genetic markers, were also generally low and/or not significantly different from zero, albeit universally higher than our estimates (significant FST estimates were less than 0.043, with the exception of FST = 0.084 between the Halda and Jamuna rivers in Alam et al., 2009; see Islam and Alam, 2004; Alam et al., 2009; Sahoo et al., 2014; Ullah et al., 2015; Qadeer and Abbas, 2017). Plausible explanations for the relatively low estimates of F_ST_ in our study include the possibly that (i) previous estimates of F_ST_ were biased upward, as the possible over representation of siblings in river spawn samples was not accounted for (Peterman et al., 2016; Wang, 2018); (ii) our estimates of population genetic parameters are themselves biased or imprecise due to the excessive purging of putative siblings, a risk particularly in the case of the Halda river from which only 17 founders with no common dummy parents were identified (Waples and Anderson, 2017); or (iii) this reflects the different properties of markers – SNPs often result in lower F_ST_ estimates than other markers (Hedrick, 2005).

The Jamuna is a tributary of the Padma river and thus the lack of substantive SNP differentiation between these rivers was not unexpected, given the potential for gene flow. In contrast, the Halda river is hydrologically and geographically isolated from the Jamuna and Padma – making genetic differentiation due to genetic drift and adaptive selection more likely, and the lack of substantive molecular marker differentiation in our and previous studies more difficult to explain. Possible explanations for a lack of molecular differentiation in markers include the large-scale translocation of fish by Government-funded seed stocking programs or the escape of hatchery-produced stock from aquaculture ponds (refer to Islam and Alam, 2004; Alam et al., 2009; Sahoo et al., 2014; Ullah et al., 2015; Qadeer and Abbas, 2017 for further discussion). However, from the perspective of genetic improvement, it should be noted that a lack of substantive molecular differentiation in putatively neutral markers does not preclude the existence of exploitable adaptive differentiation among rivers for commercially-important traits (Edelaar and Bjorklund, 2011).

Previous studies have alluded to substantial reductions in the effective population size of rohu populations, attributed to upstream dam construction and reduced flows, pollution, over fishing or over harvesting of river spawn (Alam et al., 2009; Ullah et al., 2015). However, the presence of such genetic bottlenecks in river populations may be erroneously inferred if siblings are over represented in samples (Wang, 2018), which is a risk if samples are obtained in the early life stages of highly fecund species (e.g., as fry or fertilized spawn). Accordingly, future studies examining the population genetics of rohu, and other major carp species (Hamilton et al., 2019a), should be undertaken on samples obtained from adult riverine fish.

## Conclusion

This study (i) successfully identified and characterized single nucleotide polymorphisms (SNPs) and silicoDArT markers in rohu (Hamilton et al., 2019b); (ii) identified an unexpectedly high level of sibship among breeding population founders; and (iii) broadly in keeping with previous studies, found no or low levels of divergence in SNPs among the three river populations studied. The sibling relationships identified have subsequently been used in pedigree-based genetic analyses of the WorldFish rohu breeding population to improve the accuracy of genetic parameter and breeding value estimates, and will be used in future parental selection and mate allocation to avoid inbreeding in the short and long term (Meuwissen, 1997; Visscher et al., 2002). Furthermore, a lack of strong genetic structuring among river populations is likely to simplify future genome wide association studies (GWAS; Nguyen et al., 2018b) and genomic selection.

## Ethics Statement

As stated in the Section “Materials and Methods.” In 2012, rohu spawn was collected from three major river systems in Bangladesh; the Halda, Jamuna and Padma (Ganges). At 2 years of age, approximately 140 individuals from each river were mated as the founders of a breeding population (Keus et al., 2017; Khan et al., 2018). All founders were fin-clipped and tissue samples archived as part of routine genetic improvement activities (i.e. the archiving of fin-clips from all candidate parents). These samples were used for the purpose of our study. Fish were anesthetized, with clove oil, prior to the removal, with scissors, of an approximately 2-mm-wide sample from fin extremities. Fish were then placed in recovery tanks for monitoring and only released back into ponds once they had satisfactorily recovered from anesthesia. Fish in the breeding population are managed in accordance with the Guiding Principles of the Animal Care, Welfare and Ethics Policy of the WorldFish Center (Worldfish, 2004).

## Author Contributions

MH performed the analyses and wrote the first draft of the manuscript. WM oversaw the establishment of the founder population using fish collected as part of the Aquaculture for Income and Nutrition (AIN) project. AK oversaw the generation of SNP and silicoDArT data. All authors contributed to manuscript writing and revision.

## Conflict of Interest Statement

AK is the Director of Diversity Arrays Technology (DArT) Pty Ltd., who undertook genotyping for this study on a fee-for-service basis. The remaining authors declare that the research was conducted in the absence of any commercial or financial relationships that could be construed as a potential conflict of interest.
