# Supplementary material for: Single Nucleotide Polymorphisms (SNPs) Reveal Sibship Among Founders of a Bangladeshi Rohu (Labeo rohita) Breeding Population
Source: Front Genet. 2019 Jun 19;10:597. doi: 10.3389/fgene.2019.00597 (PMC6593075; doi:10.3389/fgene.2019.00597)
Supplement: Supplementary file 1 [file Table_1.DOCX]

Supplementary Material

Single nucleotide polymorphisms (SNP) reveal sibship among founders of a Bangladeshi rohu (*Labeo rohita*) breeding population

Hamilton, M.G., Mekkawy, W., Kilian, A., Benzie, J.A.H

**Correspondence:** Matthew Hamilton ([m.hamilton@cgiar.org](mailto:m.hamilton@cgiar.org))

**Table S1.1**. Pairwise estimates of Wright’s (1965) F_ST_

| Population 1 | Population 2 | F_ST_  (95% confidence interval) |  |
| --- | --- | --- | --- |
| Halda | Jamuna | 0.0038  (0.0024 - 0.0055) |  |
| Halda | Padma | 0.0041  (0.0027 - 0.0056) |  |
| Jamuna | Padma | 0.0000  (0.0000 - 0.0005) |  |
| Global |  | 0.0012  (0.0006 - 0.0018) | |


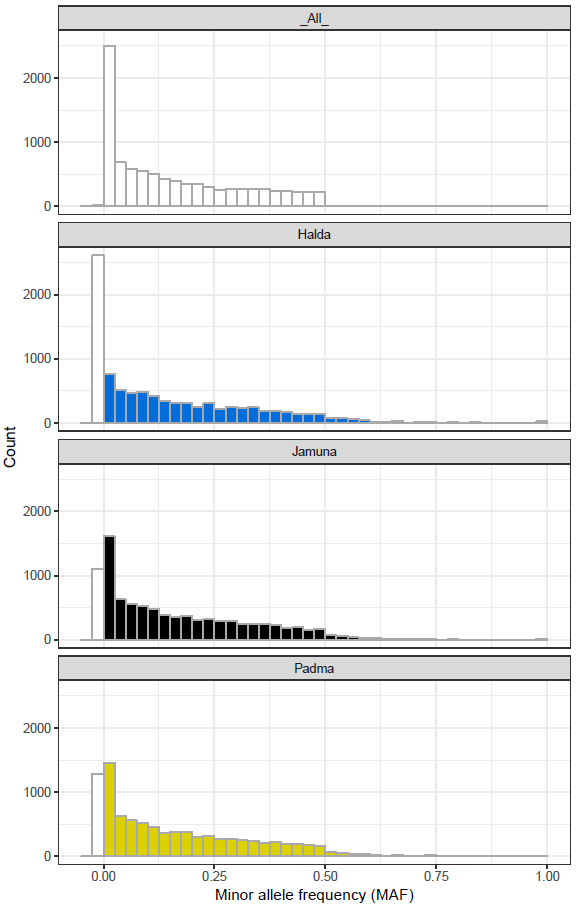

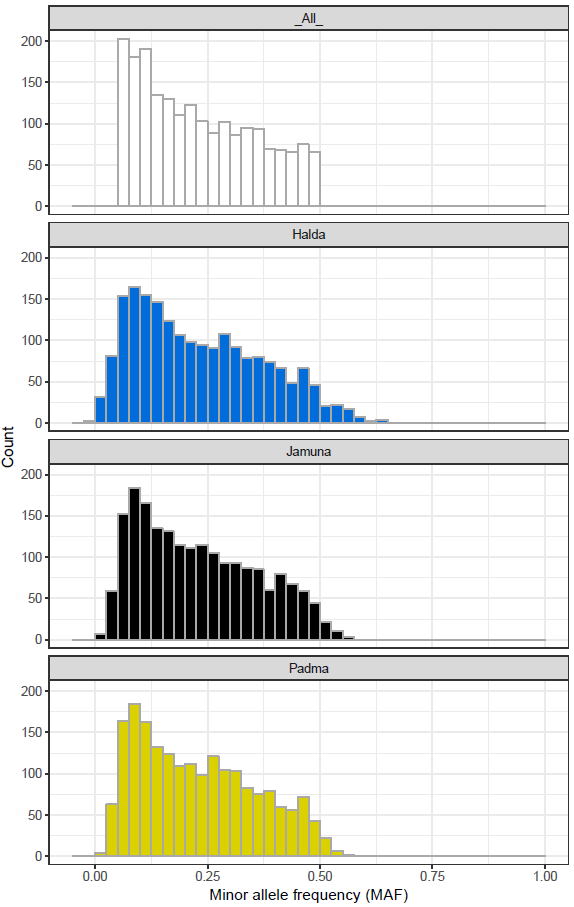


a) b)

**Figure S1.1** Minor allele frequency in across all river populations and within popuations using a) all SNP and founders prior to quality control and b) SNP and founders used in popuation genetic analyses. The minor allele for each loci was identified in the dataset containing all rivers for a and b separately. White filled bars less than zero represent SNP loci in which the minor allele was absent (i.e. MAF is exactly zero).

a)
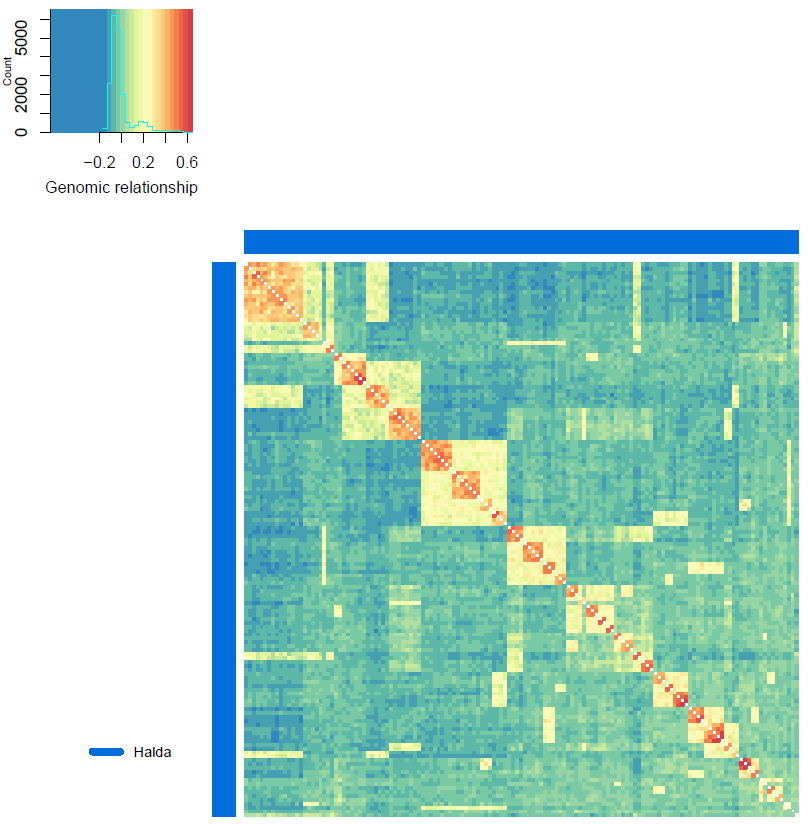


b)
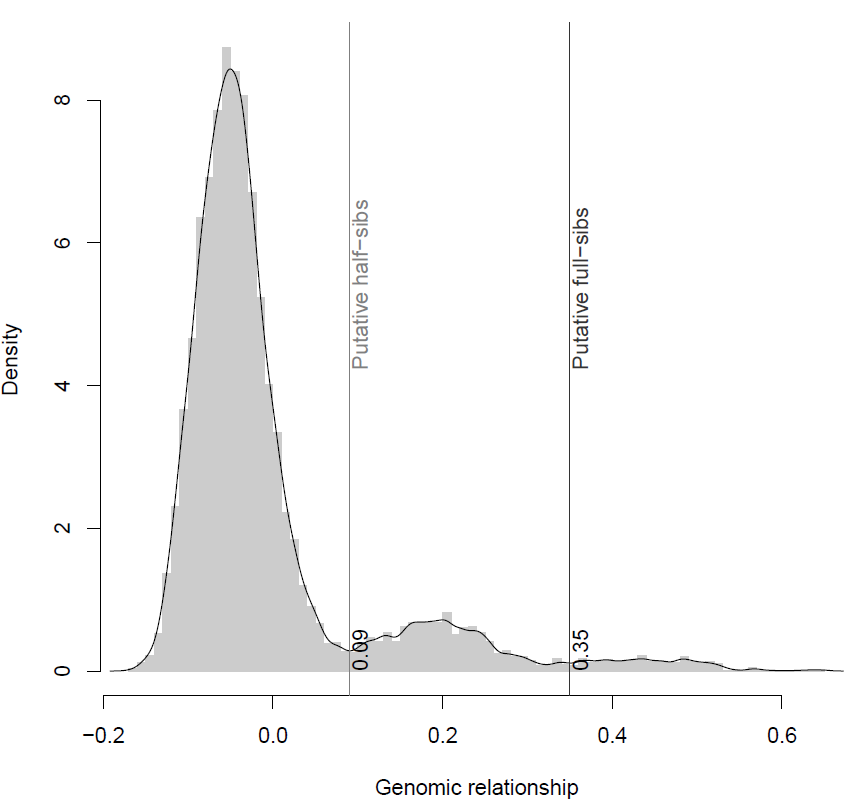


c)
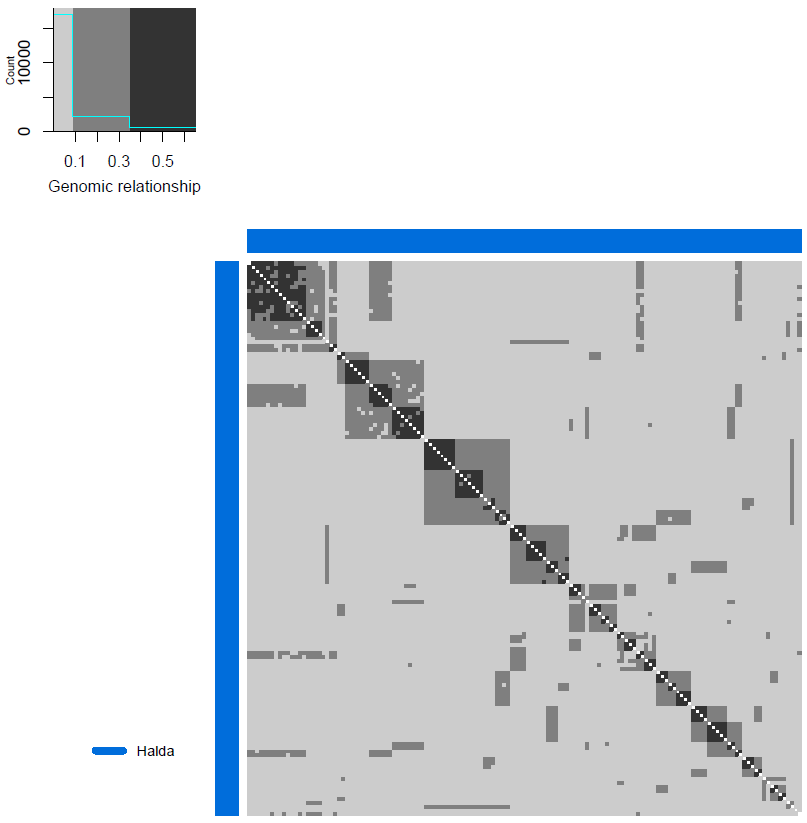


d)
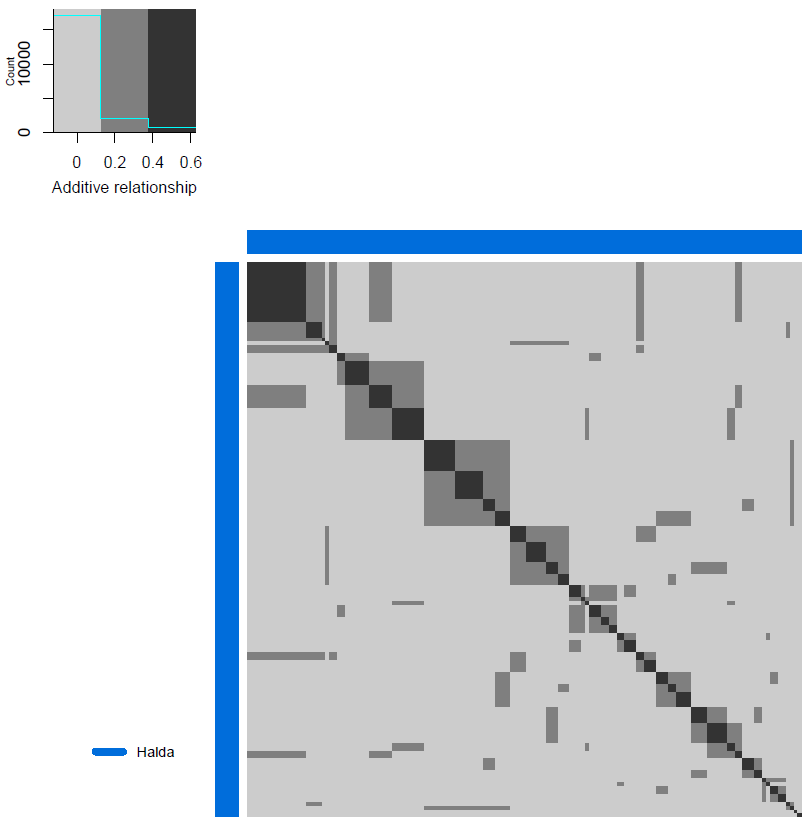


**Figure S1.2** Heatmaps of relationships and associated plots computed using Halda river data and observed allele frequencies from founders with no COLONY-assigned dummy parents in common: a) genomic relationships on a continuous scale; b) density plot of pairwise genomic relationships; c) genomic relationship on a discrete scale; d) additive genetic relationships computed from the COLONY-derived pedigree. In discrete plots, light grey cells represent putitively unrelated individuals, dark grey cells represent putative half-siblings and black cells represent putative full-siblings. Individuals are ordered according to clustering, using the ‘Ward2’ algorithm, of genomic relationships in a.

a)
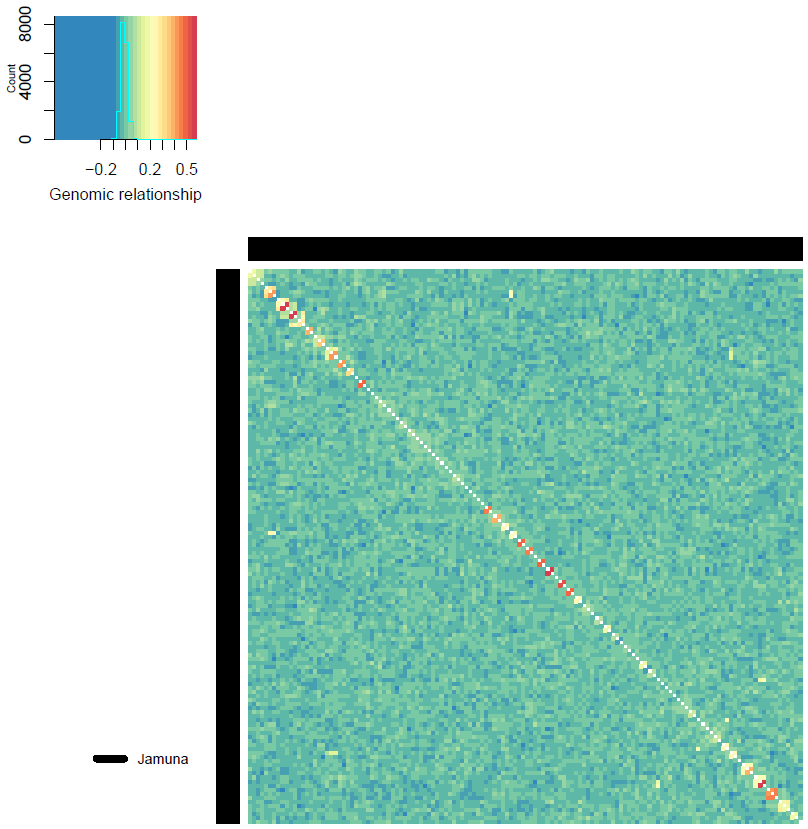


b)
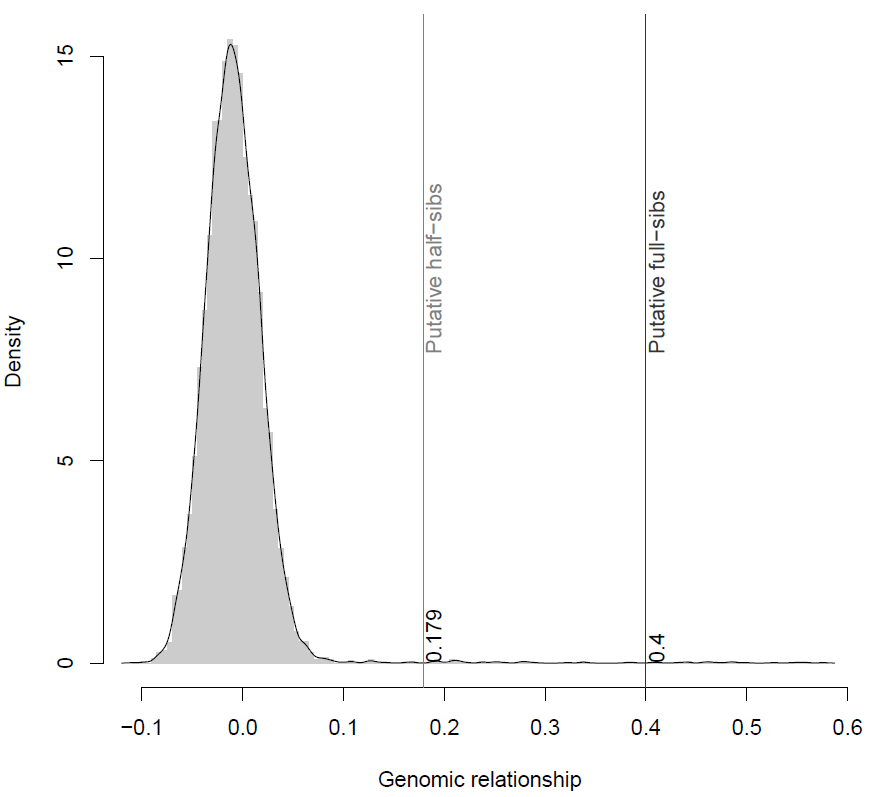


c)
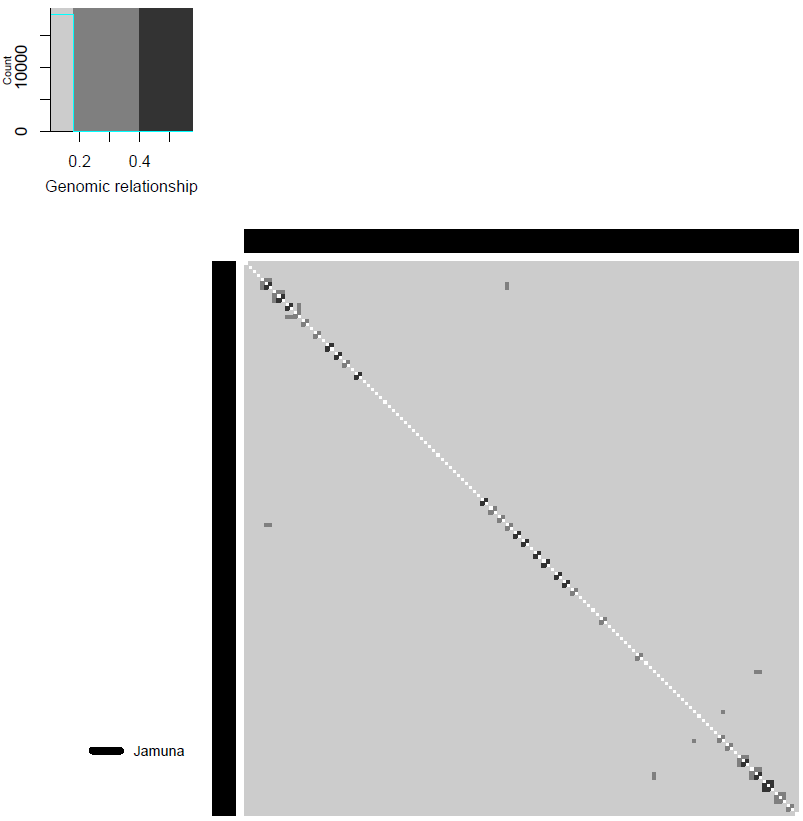


d)
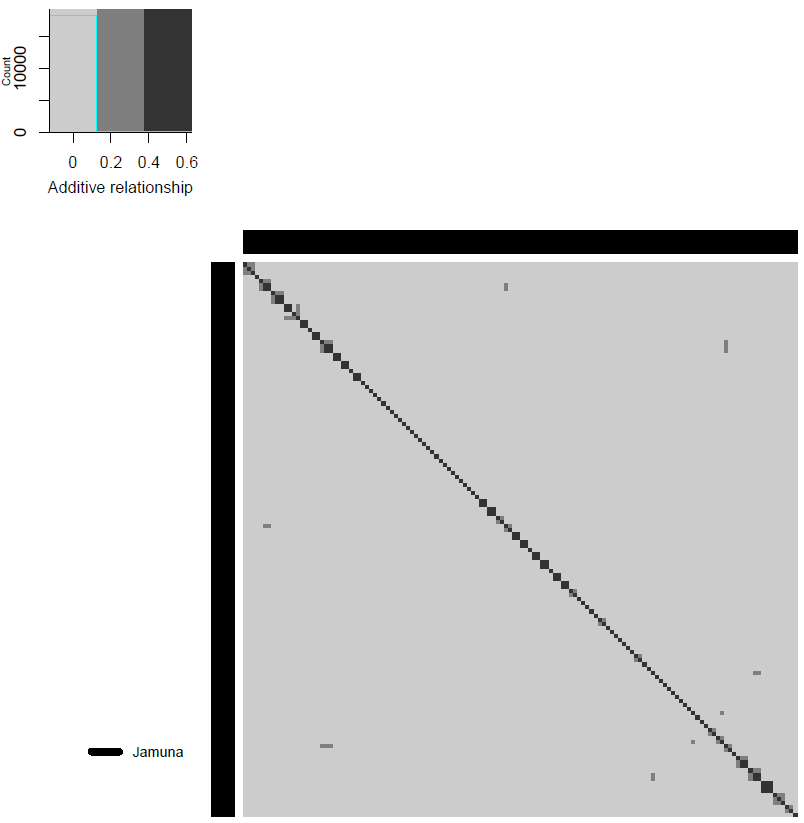


**Figure S1.3** Heatmaps of relationships and associated plots computed using Jamuna river data and observed allele frequencies from founders with no COLONY-assigned dummy parents in common: a) genomic relationships on a continuous scale; b) density plot of pairwise genomic relationships; c) genomic relationship on a discrete scale; d) additive genetic relationships computed from the COLONY-derived pedigree. In discrete plots, light grey cells represent putitively unrelated individuals, dark grey cells represent putative half-siblings and black cells represent putative full-siblings. Individuals are ordered according to clustering, using the ‘Ward2’ algorithm, of genomic relationships in a.

a)
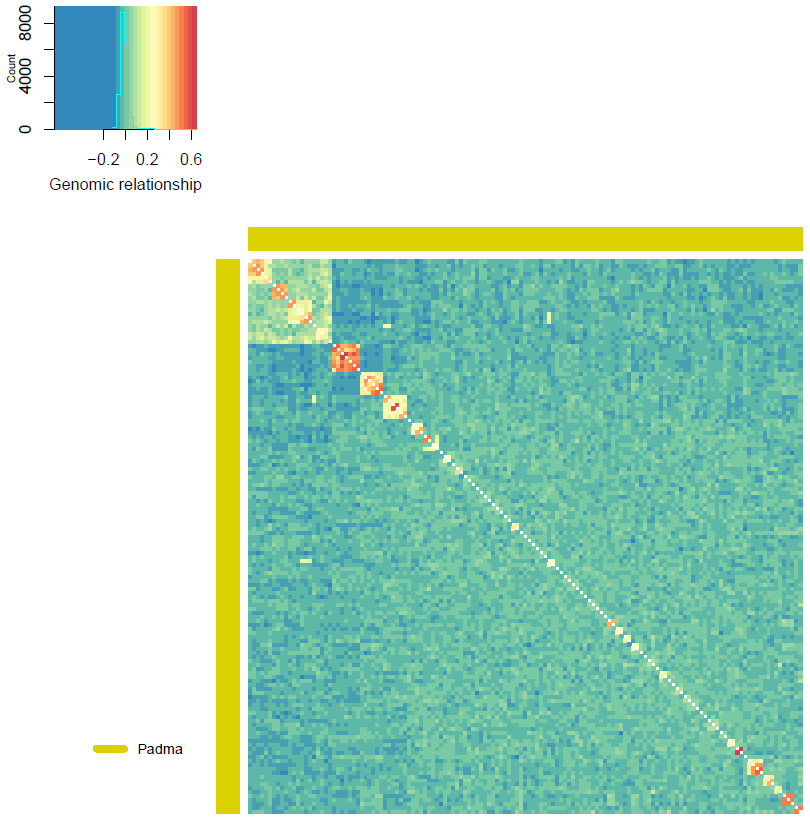


b)
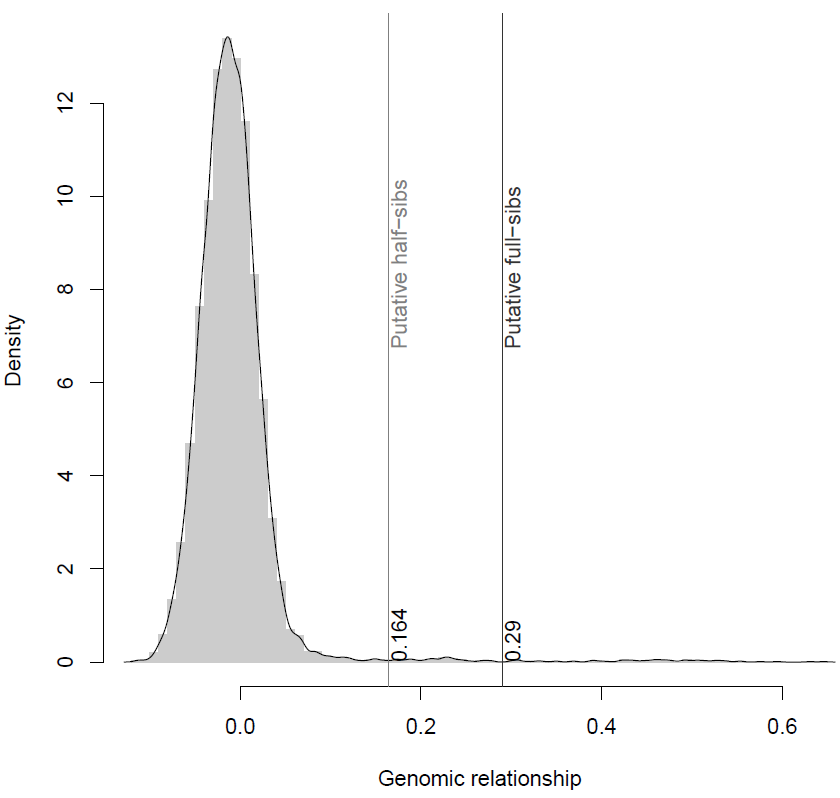


c)
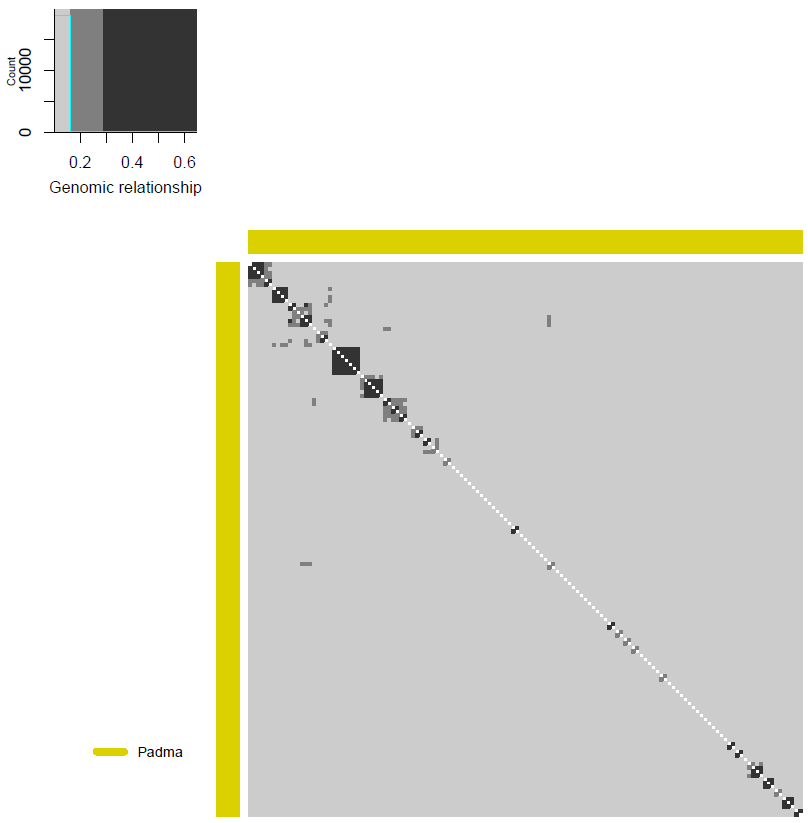


d)
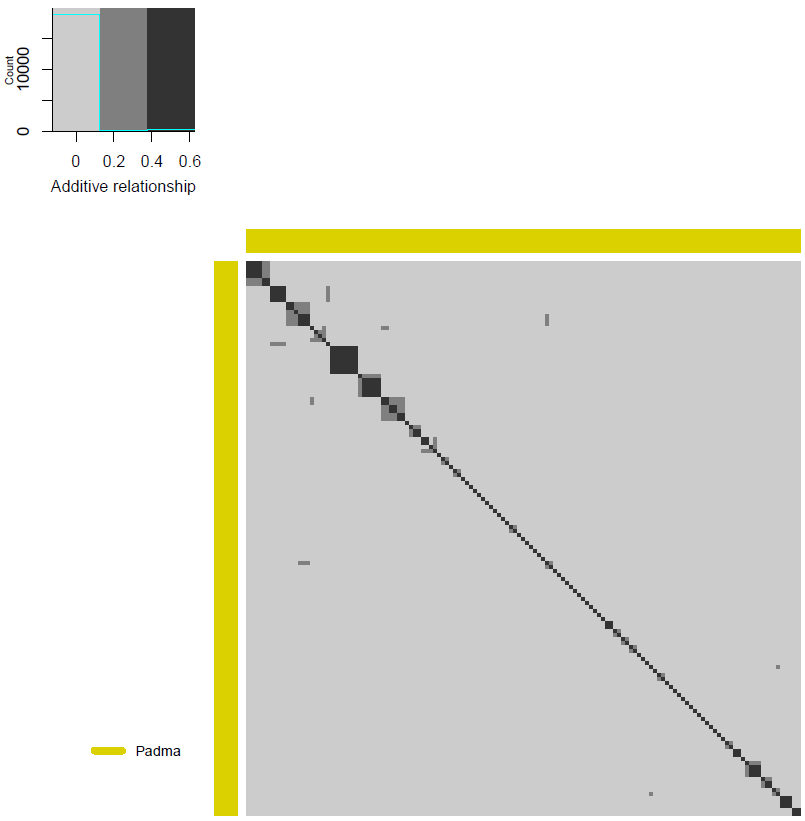


**Figure S1.4** Heatmaps of relationships and associated plots computed using Padma river data and observed allele frequencies from founders with no COLONY-assigned dummy parents in common: a) genomic relationships on a continuous scale; b) density plot of pairwise genomic relationships; c) genomic relationship on a discrete scale; d) additive genetic relationships computed from the COLONY-derived pedigree. In discrete plots, light grey cells represent putitively unrelated individuals, dark grey cells represent putative half-siblings and black cells represent putative full-siblings. Individuals are ordered according to clustering, using the ‘Ward2’ algorithm, of genomic relationships in a.


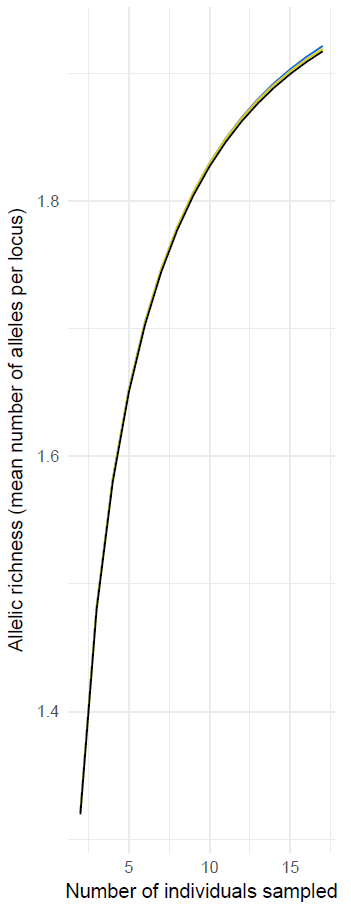

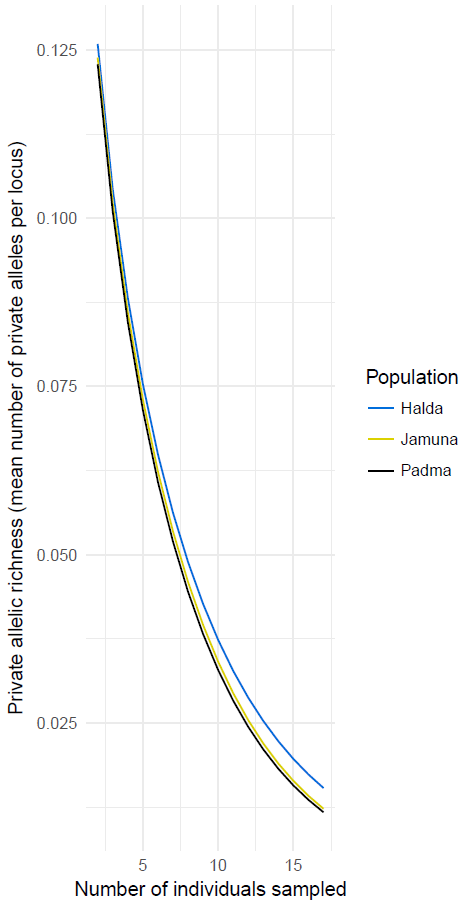


1. b)

**Figure S1.5.** The mean number of a) distinct alleles per locus and b) private alleles per locus, as functions of standardized sample size for three rivers (excluding known relatives).


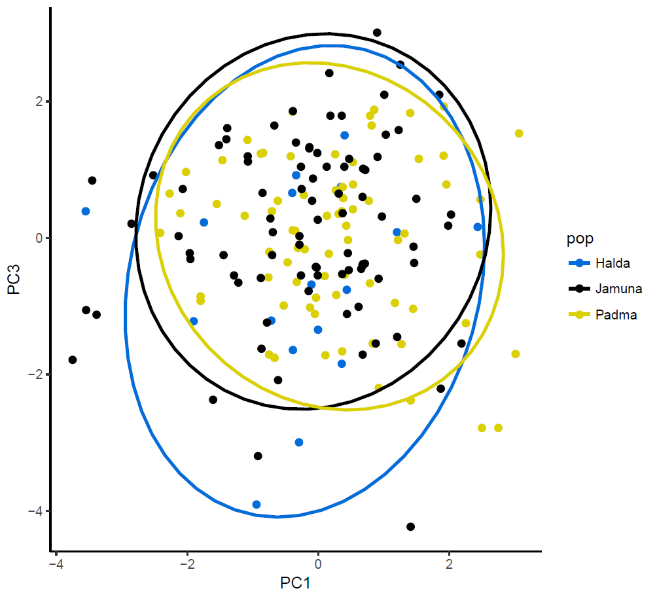

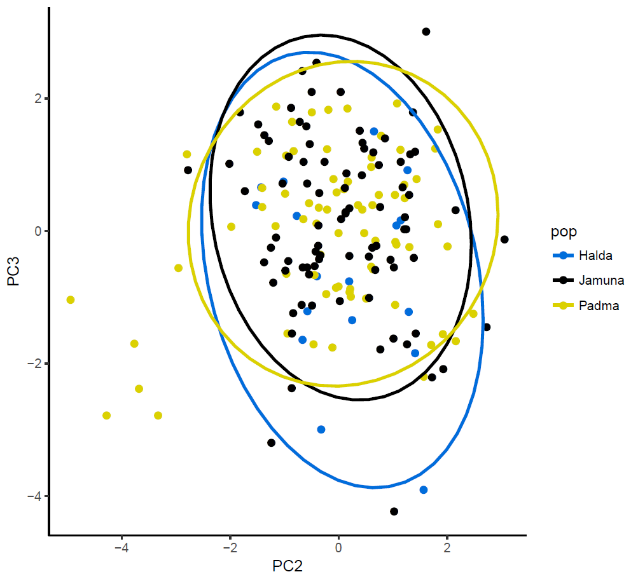

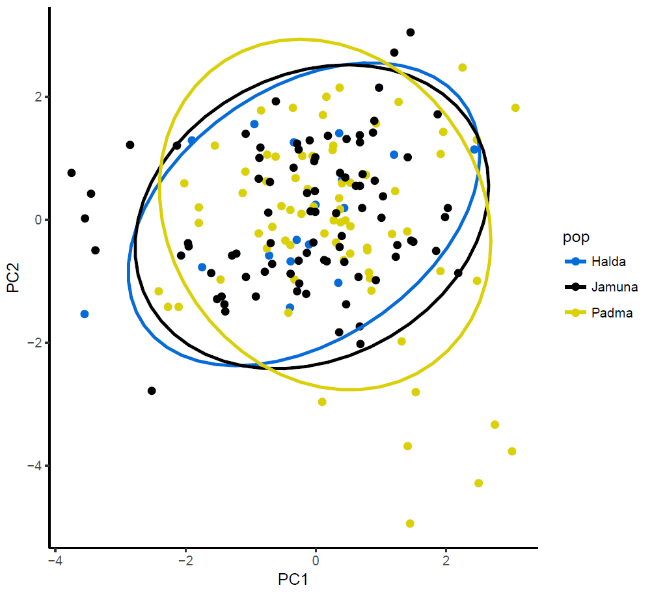


**Figure S1.6.** Scatterplots of the first three principal components.
